# Diagnostic dilemma in acute neurological presentation of spinal arachnoid cysts: A case report

**DOI:** 10.3389/fsurg.2023.1092345

**Published:** 2023-07-03

**Authors:** Swati Jain, Ira Sun, Boon Chuan Pang, Su Lone Lim, Shiong Wen Low

**Affiliations:** ^1^ Division of Neurosurgery, University Surgical Cluster, National University Health System (NUHS), Singapore, Singapore; ^2^ Division of Neurosurgery, Department of General Surgery, Ng Teng Fong General Hospital, Singapore, Singapore

**Keywords:** laminectomy, spinal arachnoid cysts, weakness, decompression, spinal cord compression diagnosis, therapy

## Abstract

**Background:**

Spinal arachnoid cysts are relatively uncommon, cerebrospinal fluid-filled sacs formed by arachnoid membranes that can be either idiopathic or acquired. The neurological presentation of these cysts is varied. Advances in imaging techniques have allowed an improved characterization of these entities and excluded other possible causes of clinical manifestation. Their presentation remains varied, ranging from pain to progressive neurological deficits. Here, we present two cases of patients with thoracic arachnoid cysts that posed a diagnostic dilemma at initial presentation because of their acute neurological deficit, and their eventual recovery after surgical intervention.

**Case description:**

The first case is of a patient with end-stage renal failure, which prevented the administration of contrast during the workup. The differential diagnosis ranged from intradural abscess to arachnoid cyst. The second patient presented with non-remitting back pain that progressed to an acute neurological deficit. Both patients recovered well after decompression of the cyst.

**Conclusion:**

The decision to intervene is still patient-dependent and based on the extent of neurological deterioration at the time of presentation due to the relatively benign nature and lack of understanding of the temporal presentation of neurological symptoms, which are rapidly and almost completely reversed after surgery. However, further studies need to be done to understand the acute presentation of these cysts, which are apparently long-standing.

## Introduction

Spinal arachnoid cysts (SAC) are relatively uncommon, cerebrospinal fluid (CSF)-filled sacs formed by arachnoid membranes that can be either idiopathic or acquired. They may be extradural, intradural, or intramedullary (1–3). They are usually asymptomatic and diagnosed incidentally on imaging during the evaluation of other unrelated presenting complaints.

Several hypotheses have been made to explain the formation of these SACs (1, 4, 5). Some authors have described cyst formation as secondary to a defect in the arachnoid mater, leading to an accumulation of CSF in the extradural or intradural space. The dynamics of CSF flow within the cyst are not well understood. The mid-to-lower thoracic levels are the most common location of these cysts, with a male predominance and presentation in the second decade of life.

The presentation of these cysts is varied, ranging from back pain, motor or sensory deficits, and incontinence to signs and symptoms of myelopathy (3, 6). Advances in imaging techniques have allowed better characterization of these entities and excluded other possible causes of clinical presentation. Neurological symptoms usually develop over a period of months. The presence of a neurological deficit is an indication for surgical intervention, which includes methods such as fenestration and excision through various posterior approaches for dorsally located arachnoid cysts (4, 6, 7).

The temporal evolution of these cysts remains unknown. The sudden, abrupt onset of neurological symptoms poses a diagnostic question to the treating physician as to whether the causative factor is the spinal arachnoid cyst. The diagnosis may be further confounded by the underlying comorbidities. Here, the authors present two cases of spinal arachnoid cysts that posed a diagnostic dilemma to the treatment team.

## Case 1

A 53-year-old woman with multiple comorbidities, including end-stage renal failure (ESRF) and poorly controlled diabetes mellitus, was admitted to the internal medicine department for fluid overload with a chest infection. She was admitted to the ICU and treated with diuresis and antibiotics. She had a persistent fever spike despite adequate treatment, and blood cultures revelaed *Staphylococcus epidermidis*. She was discharged to the general ward after 7 days, where she complained of sudden-onset fecal incontinence and leg weakness. Physical examination was consistent with bilateral lower limb weakness, with a strength grade of 2/5 from L2-S1. Her anal tone was lax, with reduced perianal sensation. No reflexes could be elicited bilaterally in the lower limbs. There was no clonus. The neurological examination was significantly affected by the ongoing sepsis, and no sensory level could be determined at that time.

**Figure 1:**
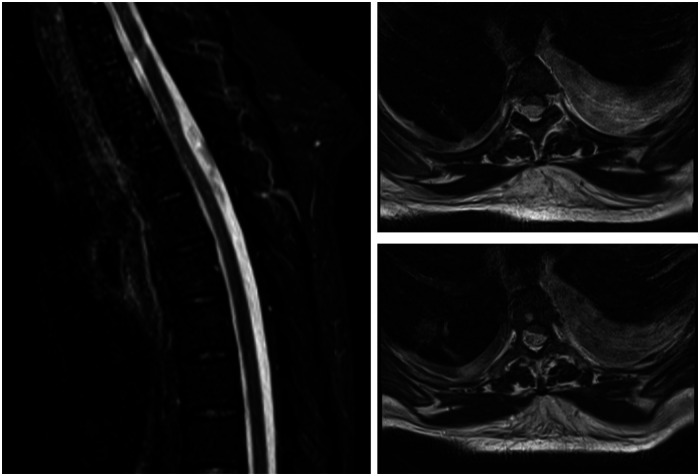
A sagittal T2 weighted and corresponding axial cross sections at two levels. MRI shows an iso- to hypointense possible intradural extramedullary lesion extending from thoracic level two to thoracic level five, which compressed the spinal cord and displaced it anteriorly.

Sagittal and axial MRI sections are shown in Figure 1. An iso- to hypointense T2 and isointense T1 intradural extramedullary lesion was seen from T2 to T5, compressing and displacing the spinal cord anteriorly. There was no associated syrinx, and no other significant findings to account for her clinical presentation. Due to her ESRF, contrast-enhanced images were not obtained. In view of the ongoing sepsis with positive blood cultures and immunocompromised status (due to diabetes and ESRF), there were concerns related to an epidural/intradural abscess. An arachnoid cyst was considered a potential diagnosis; however, it was not a top differential given the infective clinical picture.

After an extensive discussion with the patient and her family, a decision was made to proceed with surgery. She underwent T3–T5 laminectomies and exploration for a possible epidural/intradural collection. Intraoperatively, no infectious collection was seen in the epidural space. A decision was made to open the dura, where a gush of CSF was observed. No abnormal arachnoid membrane was noted. The dural opening was extended superiorly and inferiorly to ensure that no other collections were missed. The dural opening was primarily repaired. Intraoperatively, neuromonitoring did not report any changes from the preoperative baseline.

Postoperatively, there was a significant improvement in her neurological condition. The patient’s bilateral lower limb strength from L2 to S1 returned to grade 4. She was able to ambulate with assistance. She was significantly deconditioned because of her prolonged stay in the ICU and multiple comorbidities. With significant rehabilitation, she was able to ambulate with minimal assistance.

Her postoperative course was complicated by a superficial wound infection. She underwent wound debridement and vacuum-assisted closure of her wound. She declined further scans in view of her prolonged hospital stay and multiple investigations.

## Case 2

**Figure 2:**
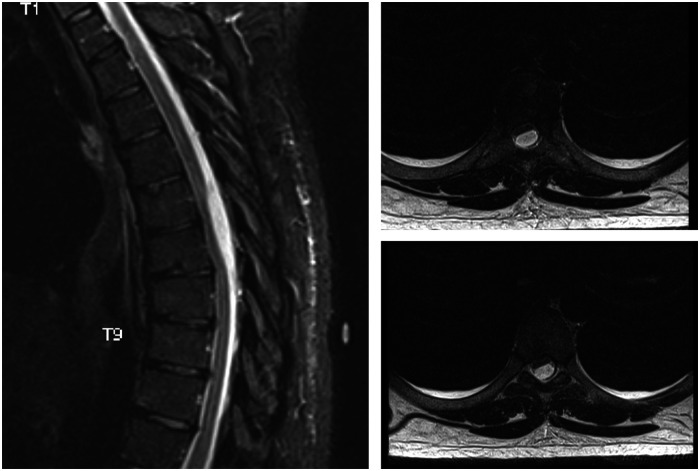
A sagittal T2 weighted and corresponding axial cross sections at two levels. MRI shows a posterior cystic lesion extending between thoracic levels six and seven, compressing the spinal cord anteriorly.

A 29-year-old man had recurrent hospital admissions with multiple episodes of right flank pain associated with a urinary tract infection. An extensive workup for his right flank pain was unremarkable. He was treated for his urinary tract infection with multiple courses of antibiotics, with a transient resolution of his symptoms. On admission, he presented with similar symptoms. However, this presentation was associated with right-sided lower-limb weakness. On clinical examination, his left lower extremity strength was found to be normal. However, the strength on the right side was as follows: L2 4/5, L3 4/5, L4 3/5, L5 3/5, and S1 4/5. There was no clonus bilaterally. The knee-jerk and ankle-jerk reflexes were hyperreflexic on the right side. Reflexes were normal on the left side. There was no objective sensory loss bilaterally. An MRI of the spine revealed a T6–T7 posterior cystic lesion compressing the spinal cord (Figure 2). No other lesions or abnormalities were identified to account for the neurological deficit. Contrast-enhanced imaging could not be obtained because the patient refused contrast-enhanced studies. However, the images were consistent with an arachnoid cyst.

**Figure 3:**
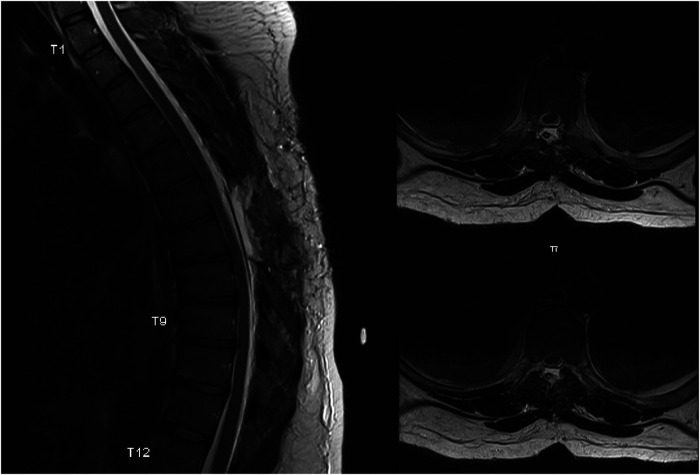
A sagittal T2 weighted and corresponding axial cross sections at two levels. MRI shows a complete resolution of the mass effect on the thoracic spinal cord.

Given the neurological deficit and persistent right-sided flank pain, a decision was made to proceed with surgery. The patient underwent a T6–T8 laminectomy and an excision of the arachnoid cyst. Intraoperative findings were consistent with a large arachnoid cyst compressing the spinal cord ventrally. The cord re-expanded after the cyst was excised. The dura defect was primarily closed. Intraoperative neuromonitoring did not reveal any abnormalities. A post-op MRI was performed, which showed complete resolution of the mass effect on the spinal cord (Figure 3).

Postoperatively, the patient recovered well. His neurological deficit resolved completely, with a strength of grade 5 on the right side. He did not have any other postoperative complications. However, there was no improvement in his flank pain. He continues to be treated with oral analgesics for this condition.

## Discussion

Nabors et al. (8) classified spinal meningeal cysts into three main categories: Type I: extradural cysts without nerve root involvement, Type II: extradural cysts with nerve root involvement, and Type III: intradural cysts. While these cysts can occur anywhere, the most common location is the thoracic spine (9). Both patients presented with an anatomic location in the thoracic spine consistent with the published literature.

The pathogenesis of these spinal arachnoid cysts is not well understood. Cyst expansion due to various mechanisms may lead to increasing pressure on the spinal cord, leading to the presentation of neurological symptoms. Various theories, such as the one-way ball mechanism, the osmotic gradient between the subarachnoid space and the cyst, and active secretion by the cyst wall cells, have been proposed to explain why the cysts become symptomatic (4, 6, 10, 11). In our two patients, there was no history of spinal trauma or intervention to suggest an iatrogenic cause of the cysts. The patients presented with a neurological deficit, in addition to back pain in the second case.

Due to the lack of previous imaging, it was not possible to determine a temporal change in the size of the cyst or an increase in mass effect leading to their acute presentation. The neurological examination findings were consistent with the known myriad presentations of this condition. In a study by Bassiouni et al. (12), 47.6% of patients presented with thoracic myelopathy with slowly progressive paraparesis. Interestingly, the study showed that patients with myelopathy had a longer duration of symptoms compared to those presenting with radicular symptoms or back pain. However, in our two cases, both patients had a relatively acute presentation of a neurological deficit. Multiple studies have shown a direct increase in intracranial pressure with ongoing dialysis (13–15). Whether dialysis contributed to changes in the intraspinal CSF dynamics that led to acute deterioration in the first patient is a possible postulate for this acute presentation.

The characteristic image finding on MRI is that of a non-enhancing extramedullary loculated CSF collection displacing the cord (3, 5, 6). The signal characteristics are the same as those of CSF and are non-enhancing when contrast-enhanced images are taken. If myelography is performed, it will usually show a contrast-filling defect in the cyst and effacement of the subarachnoid space outside the cyst. Bony changes may be seen due to the long history of the arachnoid cyst. The first patient presented with a diagnostic dilemma because of her ongoing bacteremia and her inability to proceed with a contrast-enhanced study due to her underlying end-stage renal failure. With the acute onset of a neurological deficit, ongoing bacteremia, and the presence of a spinal cord lesion, an epidural or intradural abscess could not be ruled out on the basis of imaging alone. Hence, a decision was made to proceed with an urgent surgical procedure for both diagnostic and therapeutic purposes. The intraoperative findings of an arachnoid cyst continue to highlight its varied presentation. There are no studies in the literature that have shown whether systemic sepsis can lead to acute decompensation in a patient with a spinal arachnoid cyst. Sepsis leads to changes in overall body fluid balance, especially in a patient with underlying renal failure. Whether these fluid changes may have altered the CSF flow mechanism within the cyst, leading to neurological deterioration, is another possible hypothesis for this patient. No dural defect was found intraoperatively to explain the sudden increase in cyst size.

Treatment for incidental SACs remains controversial, with a conservative approach being preferred due to the unknown natural history of this entity when the patient is asymptomatic (16–18). Laminectomy and excision of the cyst remain the treatments of choice in symptomatic patients. Some authors have suggested preoperative aspiration of the cyst under radiologic guidance, with the caveat that this cyst will recur if there is persistent communication with the subarachnoid cyst. Multiple series have shown excellent outcomes after cyst resection. French et al. (9) showed in their study of 10 patients that either fenestration or complete excision resulted in a complete resolution of pain and improvement in neurological function. Their study showed that patients who had pain as their primary presenting complaint had excellent outcomes. In contrast, studies by Petridis (19) have shown that hypesthesia and neuropathic pain are less likely to resolve after surgery. Both of our patients showed remarkable postoperative recovery. In our second patient, there was an immediate improvement in his neurological deficits, but the pain persisted despite an adequate titration of analgesia. The patient's neuropathic pain was long-standing and was attributed to causes such as recurrent UTIs. Duration of pain and timing of intervention may play a role in the eventual recovery; however, no studies are available to understand this temporal relationship because of the rarity of this phenomenon.

## Conclusion

Our report highlights the myriad presentations of the rare entity known as spinal arachnoid cysts. Both patients presented with acute deterioration and weakness in their lower limbs. The decision to intervene is still patient-dependent and based on the extent of neurological deterioration at the time of presentation due to the relatively benign nature of this phenomenon and lack of understanding of the temporal presentation of neurological symptoms, which are rapidly and almost completely reversed after surgery. However, further studies need to be done to understand the acute presentation of these cysts, which are apparently long-standing.

## Data Availability

The original contributions presented in the study are included in the article/Supplementary Material; further inquiries can be directed to the corresponding author.
